# A study on the current state and equity level of the health promotion service demands among older adults in China

**DOI:** 10.1186/s12939-023-01882-x

**Published:** 2023-04-13

**Authors:** Weicun Ren, Xiwang Ma, Clifford Silver Tarimo, Yiqing Xing, Xinyuan Lv, Zhang Liang

**Affiliations:** 1 School of Political Science and Public Administration, Wuhan University, Wuhan, China; 2 College of Public Health, Zhengzhou University, Zhengzhou, China; 3 Department of Science and Laboratory Technology, Dares Salaam Institute of Technology, Dares Salaam, Tanzania

**Keywords:** Health promotion service, Demand, Older adult, Equity, TOPSIS

## Abstract

**Background:**

Meeting the demands of older adults for health promotion services (DOAHPS) is essential for maintaining their health and enhancing their quality of life. The purpose of this study was to construct a model for evaluating DOAHPS to quantitatively evaluate the current state and equity level of DOAHPS in China, as well as to explore the main factors affecting DOAHPS’ current state and equity level.

**Methods:**

This study analyzed the DOAHPS data from the "Survey on Chinese Residents' Health Service Demands in the New Era", which included 1542 older adults aged 65 and older. Relationships between evaluation indicators of DOAHPS were explored using Structural Equation Modeling (SEM). The Weighted TOPSIS method and Logistic regression (LR) were used to analyze the current state and factors impacting DOAHPS. The equity level of DOAHPS’ allocation among different older adult groups and its influencing factors were determined using the Rank Sum Ratio (RSR) method and T Theil index.

**Results:**

The evaluation score for DOAHPS was 42.57 ± 1.51. Health status, health literacy and behavior were positively correlated with DOAHPS (*r* = 0.40, 0.38; *P* < 0.05). The LR results revealed that the most significant determinants of DOAHPS were sex, residence, education level and pre-retirement occupation (all *P* < 0.05). The number of older adults with very poor, poor, general, high and very high level health promotion service demands accounted for 2.27%, 28.60%, 53.05%, 15.43% and 0.65%, respectively. The total T Theil index of DOAHPS was 2.7433*10^–4^, and the intra-group difference contribution rate exceeded 72%.

**Conclusions:**

Compared to the maximum level, the total DOAHPS level was found to be moderate, although the demands of urban seniors with higher levels of education may be substantially greater. The observed inequities in the allocation of DOAHPS were primarily related to differences in education level and pre-retirement occupation within group. To better address health promotion services for older adults, policymakers could target older males with low education who reside in rural regions.

## Introduction

The organ function and general health of the human body change with age, showing a gradual or rapid decline trend after adulthood [1]. China's guidelines for the protection of the rights and interests of older adults classifies those over 60 as older adults based on changes in bodily function [2]. However, the “Principles and Recommendations for a Vital Statistics System” believes that the definition of the older adult over 65 is regarded as more suitable for today's social development and demographic trends [3]. In 2020, the proportion of the population over the age of 65 in Hubei, Guizhou and Guangzhou provinces has all exceeded 10%, and the dependency coefficient of older adults ranges from 11.82% to 21.11% [4]. As older adults reach retirement age, their physical and mental health deteriorates, making health a critical concern [5]. The health state of older adults is not only affected by their own genetic factors, but also closely related to their living habits, medical services, utilization of health promotion services (HPS), just to mention a few [6]. Among these, HPS are necessary measures that people propose to improve and protect their health and well-being [7].

The utilization of HPS by older adults can help improve their quality of life and life expectancy, as these services identify treatable health problems and manage life-threatening diseases [8]. Therefore, understanding the demands of older adults for health promotion services (DOAHPS) can aid in mitigating the adverse effects of aging across societies [9]. Studies have shown that 21.70% of the high mortality rate occurs due to delays in HPS use, and this increase represents a negative effect of lower HPS utilization given the rising rate of chronic diseases [10]. In developing countries, rural communities, health promotion facilities have faced a number of barriers including lack of human and material resources, poor roads, and poor transportation systems [11]. In 2019, the release of the "National Medium and Long-Term Plan for Actively Responding to Population Aging" indicated that, China had achieved a strategic shift from treatment-centered to health-centered, and actively promoted integrating health into all policies [12]. Yan YP et al. [13] believed that the current effective DOAHPS in China was insufficient, while a study by Pan X et al. [14] found that meeting DOAHPS had an positive impact on their quality of life.

According to the three levels of HPS in public health theory, DOAHPS can be reflected by the comprehensive demands of self-health care, health education and health care [15], and mainly depends on their health status, health literacy and behavior, preventive care services utilization, et al. [16]. Individuals with a medical history, and limitations in activities of daily life may be more concerned about changes in their health status, and subsequently, make more or less use of HPS [17]. However, different scholars have found that there was a large uncertainty between health status and the utilization of HPS. Some studies have indicated a positive correlation between the two [18], but others have demonstrated a negative correlation [19]. The literature showed that good health literacy and behavior were independent protective factors affecting residents' demand for HPS [20]. Diao XB [21] found that improving residents' awareness of health promotion and developing regular HPS can help increase residents' demands and ensure better development of HPS. A study of retired college teachers found that their healthy behaviors were significantly associated with the demand for HPS [22]. At the same time, the current utilization of preventive care services by older adults also affects their future demands for HPS [23, 24].

In addition, studies have shown that the DOAHPS also depends on a variety of factors, such as age, residence, pre-retirement occupation, education level, and purchase of health insurance [13]. The effect of age on HPS utilization is still controversial and debatable. Iris C et al. [25] argued that as people age, they receive fewer and fewer benefits from HPS, which leads to a decline in HPS usage, but Hsieh et al. [26] held contrasting views. In China, the demand and utilization of HPS among urban older adults was 1.3 times that of the rural older adults [27]. Zhu Y et al. [28] believed that the demand for HPS for older adults with higher occupational, medical insurance, and education levels was also higher. Nicholas JA [29] found that the passage of the US Patient Protection and Affordable Care Act enhanced access to HPS by reducing out-of-pocket costs for older adults and increasing reimbursement to healthcare providers. At the same time, there are differences in HPS demands of older adults by sex [30].

In addition to the two dichotomous variables, sex and residence, the DOAHPS tends to show different trends in response to changes in various multi-categorical factors such as age, education level, and type of medical insurance. However, this change is usually nonlinear and related to single or multiple factors [31]. In rural China, for instance, the demand and utilization of HPS among those aged 80 and beyond was 1.94 times that of those aged 60 to 69, although there was no significant difference in the demand and utilization of HPS among those aged 70 to 79 [27]. One study also suggested that HPS should be provided equally to older adults at all levels of social classes, with priority given to those most in demand [32]. As the number and proportion of older adults continue to rise, improving the equitable allocation of demand-based HPS across different demographic groups of older adults is critical to delivering efficient services with limited resources.

To sum up, numerous studies have been conducted on HPS for older persons, but limited studies have examined the demand and equity level for the allocation of older adults for HPS, as well as the factors that influence demand and equity. The originality of this study lies in its objective construction of an evaluation model for DOAHPS, its quantitative analysis of the current state and allocation equity level of DOAHPS, and its discussion of the influencing factors and their relationship to the current state and equity level.

To help develop effective strategies for the provision and utilization of HPS for older adults and to improve the efficiency of services, this study used Structural Equation Modeling (SEM), Weighted TOPSIS method, and Logistic regression (LR) model to explore the components, states, and influencing factors of DOAHPS. The Rank Sum Ratio (RSR) method and T Theil index were used to analyze the equity level in the allocation of DOAHPS and the main drivers of inequality.

## Materials and methods

### Data sources

**Fig. 1:**
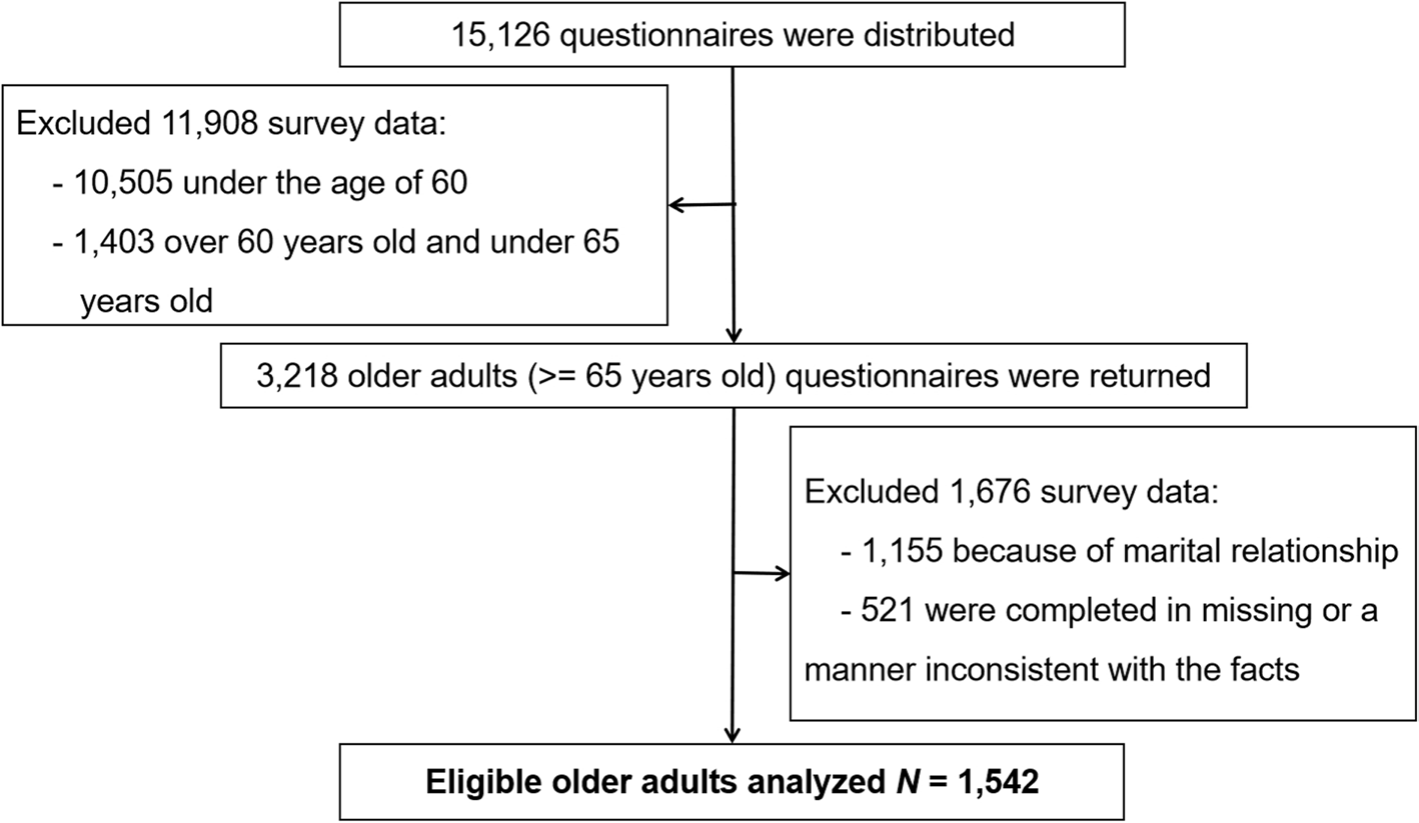
Schematic diagram for sample size determination

The DOAHPS data was retrieved from the "Survey on Chinese Residents' Health Service Demands in the New Era", which was carried out in July–August 2018 [33–35]. This survey adopts a multi-stage stratified random sampling method to select the sample into three stages. In the first step, two counties (Sinan and Dangyan) and two districts (Futian and Xiling) were selected from rural and urban China. In the second step, five towns (streets) were chosen at random in each county (district) based on their proximity to the county hospital. Six villages (communities) were then chosen at random in each town (street) based on their proximity to the township (street) hospital, for a total of 30 villages (communities) in each county (district). In each village (community), 40 families were sampled in three stages, and more households were added if rejections or closures occurred. In the sample size calculation, the design effect was set at 2.5, with an acceptable error for a significant level of 0.05 and the prevalence of chronic diseases in the population set at 21.34%. The minimum sample size was calculated to be 3,600 individuals in 30 villages (communities) in each study center, and a total of 15,126 questionnaires were collected. Participants in this study were restricted to older adults over the age of 65 (3,218 individuals in total). In order to exclude the influence of family circumstances on the study's findings, just one older individual was randomly selected from each household. Eventually, a total of 1,542 participants were registered and evaluated, excluding those whose questionnaires were incomplete or inconsistent with the facts (Fig. 1). Details of the sampling specific to an individual district (county) can be accessed in previous published studies [33–35]. All sampled households were picked systematically from the village committee's household registry, and all the members of the sampled families were investigated. Inclusion criteria included familiarity with the fundamental content of residents' health services, willingness to engage in the survey, and registration in the local village committee (neighborhood committee) household register.

The survey was conducted by a group of undergraduate and graduate students in preventive medicine and other specialties. All interviewers had received specialized training prior to conducting the investigation. A pretest was undertaken to verify the clarity and consistency of the responses in order to assure data quality. All respondents were told of the survey's purpose in advance and gave their consent to participate. Immediately after the interview concluded, the investigators completed the form. On the day that the questionnaire was collected, investigators submitted their responses, which were then checked by an experienced supervisor. Questionnaires that did not match the facts or were missing and could not be supplemented were deemed ineligible. Two individuals independently entered data into Epidata 3.1 software, comparing the two files for consistency verification and used frequency checks to identify missing values and outliers. After evaluation by the Ethics Committee of Tongji Medical College of Huazhong University of Science and Technology, it was concluded that the content and procedures of this study met the ethical requirements of international and national biomedical research (IORG number: IORG0003571).

### Evaluation model

The evaluation indicators used in this study were derived from the questionnaire of the "Survey on Chinese Residents' Health Service Demands in the New Era". The questionnaire includes five parts: "Basic family information form", "Personal general information form", "Self-rated health status form", "Prevention and health care status form" and "Medical care services utilization status form", and has good reliability and validity [33, 34]. The indicators used in this study mainly included the personal general information, self-rated health status, demand and utilization of HPS of older adults, and the specific indicators and their related meanings of DOAHPS were shown in Table 1 [16, 18, 21, 25–29].

**Table 1:** The meaning and assigned value of indicators

Variable category	First-level indicators	Secondary indicators	Meaning	Assigned value
Functional independent variables	Health status (Y1)	Behavioral capacity (x 1)	Level of behavioral ability such as autonomous walking and recognizing orientation	0-2^a^
		Self-care (x 2)	Level of self-care ability such as washing, dressing, and using the toilet	0–2
		Daily activities (x 3)	Level of ability to perform daily activities such as work, reading, or household chores	0–2
		Physical pain (x 4)	The extent to feel pain or discomfort	0–2
		Anxiety or depression (x 5)	The severity of conditions such as anxiety or depression	0–2
	Health literacy and behavior (Y2)	Communication (x 6)	Ease of communication with providers when accessing health or preventive care services	0-4^b^
		Health information (x 7)	The extent to actively read information about health or wellness	0–4
		Information utilization (x 8)	The extent to which change one's own lifestyle as directed by health or wellness information	0–4
		Smoking^c^ (x 9)	Whether to smoking (smoking refers to those who have smoked continuously or accumulatively for 6 months or more)	0–2
		Drinking^c^ (x 10)	Whether to drink alcohol (drinking refers to drinking at least once a week for six months or more)	0–2
		Physical exercise (x 11)	Average weekly physical activity in the past 30 days	0-7^d^
	Preventive care services utilization (Y3)	Health checkup (x 12)	Whether to have participated in regular health examinations (non-entry physical examinations, non-disease-related examinations)	0-1^e^
		Family doctor (x 13)	Whether there is a contract with a family doctor	0–1
		Health publicity (x 14)	Have you received health publicity/guidance provided by primary medical institutions, et al.? (Excluding disease follow-up and follow-up)	0–1
Outcome variable	Health promotion service demands (Y4)	Health guidance (x 15)	The degree of demand for the method and intensity of fitness and health care provided guide by community/village clinics, et al	0–4
		Health education (x 16)	The degree of need for information such as health care knowledge and healthy life guidance provided by the Internet and other channels	0–4
		Health care (x 17)	The degree of need for regular medical check-ups and home care services	0–4

^a^0 means that the function or ability of older adults was impaired or extremely low, and 2 means that the function or ability of older adults was normal; ^b^0 means "not at all" or "not needed at all", 4 means fully met or required. The higher the score, the stronger the behavioral ability/neediness of older adults. ^c^0 means smoking or drinking, 1 means quitting smoking or drinking, 2 means no smoking or drinking; ^d^0 means not exercising, 1–6 means the average frequency of physical exercises per week was 1 to 6 times, and 7 means the average number of physical exercises per week was 7 times or more; ^e^0 means "No", 1 means "Yes"

#### Structural Equation Model (SEM)

**Fig. 2:**
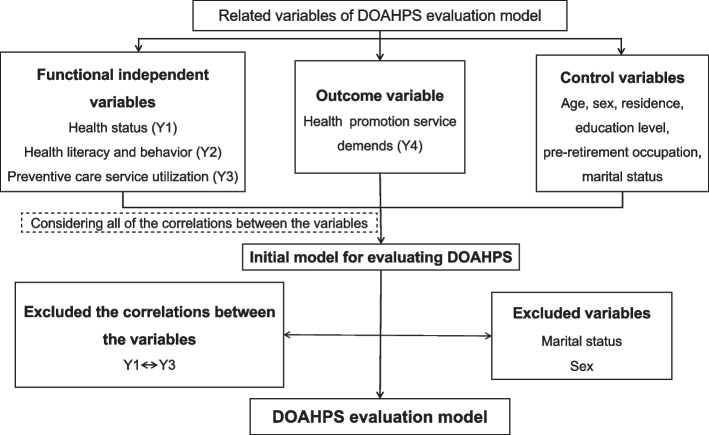
Schematic diagram of variable selection. DOAHPS: Demands of older adults for health promotion services

**Fig. 3:**
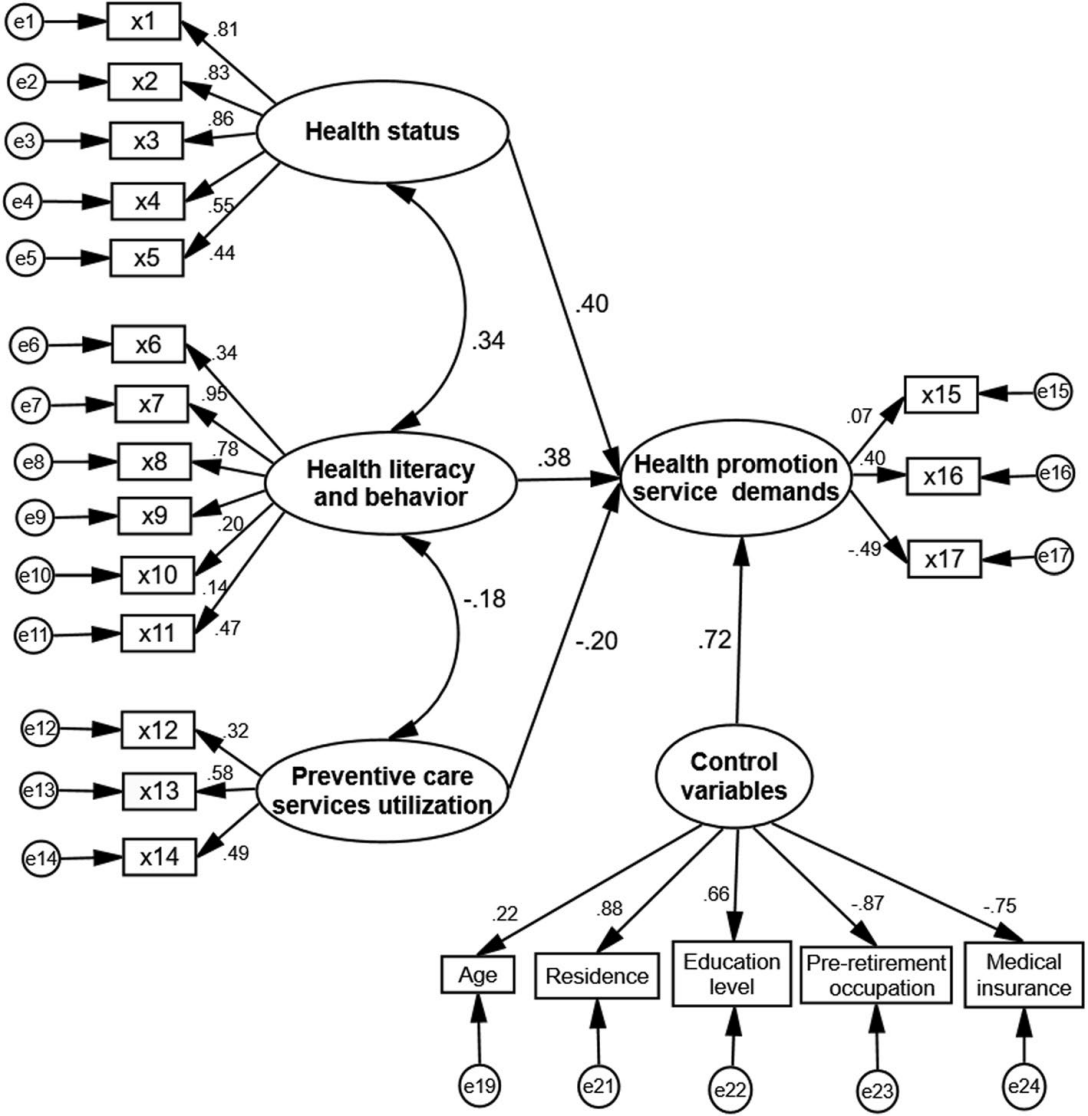
Evaluation model of the demands of older adults for health promotion services

SEM is a method for establishing, estimating and testing a causal relationship model, which includes both observable explicit variables and latent variables that cannot be directly observed [36]. In this study, SEM was used to analyze the relationship among the evaluation indicators of DOAHPS. On the basis of current relevant theoretical and practical investigations, an initial model for measuring DOAHPS was developed, incorporating all relationships between the evaluation indicators. The model also includes control variables such as age, sex, residence, education level, pre-retirement occupation, marital status, and medical insurance of older adults [13, 25–30]. The findings of the relationship between the indicators showed that sex and marital status were not statistically significant (*P* > 0.05). As a result, they were deleted (Fig. 2). The evaluation model of DOAHPS, including the correlation coefficient (*r*) between the indicators used in this study, is shown in Fig. 3.

The goodness of the fit index, incremental fit index, standard fit index, and comparative fit index of the DOAHPS evaluation model were all greater than 0.70, indicating the optimal fitting effect, but the chi-square degree of freedom ratio was greater than 3. The model is acceptable, but the overall significance is not too high. This situation could be related to a large amount of data in the model and the addition of more control variables.

#### Weighted TOPSIS method

The TOPSIS (Technique for Order Preference by Similarity to Ideal Solution) method is a common method in multi-objective decision analysis of finite schemes in systems engineering, and is widely used in many fields [37]. Based on the normalized original data matrix, it decomposes the positive ideals in the finite scheme into a space. The scheme to be evaluated can be regarded as a certain point in space, from which the Euclidean Distances *Di*^+^ and *Di*^*−*^ between this point and the positive ideal solution can be obtained. Thereby, the relative closeness value (*ζ*) of the scheme is evaluated and the positive ideal solution is obtained, and the pros and cons of the scheme are evaluated according to the value of *ζ* [38]. In this study, the Weighted TOPSIS method based on the reward and punishment function was used to evaluate DOAHPS:

$${d}_{ij}=\frac{1}{{x}_{ij}}$$

$${W}_{ij}=\frac{{d}_{ij}}{\sum\limits_{j=1}^{14}{d}_{ij}}$$

Among them, *x*_*ij*_ (*i* = 1, 2, …, 1542; *j* = 1, 2, …, 14) is the evaluation value of each indicator; *d*_*ij*_ represents the reward and punishment function value; *W*_*ij*_ represents the weight value of each indicator. Because the original data has been set as high-quality indicators before the analysis, the original data matrix is normalized:

$${Z}_{ij}={x}_{ij}/\sqrt{\sum_{i=1}^{n}{x}_{ij}^{2}}$$

Determine the optimal vector Z^+^ and the worst vector *Z*^*−*^:

*Z*^+^  = (*Z*_*i1*_^+^*, Z*_*i2*_^+^*,*⋯*, Z*_*im*_^+^)^T^;* Z*_*ij*_^+^  = max [*Z*_*ij*_];* i* = (1, 2,⋯, 1542), *j* = (1, 2,⋯, 14).

*Z*^*−*^ = (*Z*_*i1*_^*−*^*, Z*_*i2*_^*−*^*,*⋯*, Z*_*im*_^*−*^)^T^; *Z*_*ij*_^*−*^ = min [*Z*_*ij*_]; *i* = (1, 2,⋯, 1542), *j* = (1, 2,⋯, 14).

*Z*_*ij*_^+^ and *Z*_*ij*_^*−*^ represent the maximum and minimum values of the *i-th* evaluation object in the *j-th* index, respectively.

$${D}_{i}^{+}=\sqrt{\sum_{j=1}^{m}{\left[{W}_{j}\left({Z}_{ij}-{Z}_{ij}^{+}\right)\right]}^{2}}$$

$${D}_{i}^{-}=\sqrt{\sum_{j=1}^{m}{\left[{W}_{j}\left({Z}_{ij}-{Z}_{ij}^{-}\right)\right]}^{2}}$$

$${C}_{i}=100\frac{{D}_{i}^{-}}{{D}_{i}^{-}+{D}_{i}^{+}}$$

*D*_*i*_^+^ and *D*_*i*_^*−*^ represent the Euclidean distance between each evaluation object and the optimal solution and the worst solution, respectively. The value range of *C*_*i*_ is 0–100, and the value of *C*_*i*_ is close to 100, indicating that the evaluation object is closer to the positive ideal solution. According to the size of *C*_*i*_, the pros and cons of each evaluation object are sorted, and the larger the value of *C*_*i*_, the better the comprehensive level.

#### Rank Sum Ratio method and T Theil index

The Rank Sum Ratio (RSR) method is the one that uses the concept and method of parametric statistical analysis to study the allocation of RSR, and uses the RSR value to directly rank and classify the pros and cons of the evaluation objects [39]. The RSR method reveals the combination of modern nonparametric statistics and classical parametric statistics, making the two complement each other. When the RSR method is used to evaluate multiple evaluation objects, there is usually the ranking and classifying method and the credible interval method. This study mainly uses the ranking and classifying method [40]. By calculating the RSR value of each evaluation object, the method can directly rank the evaluation objects, and can further check whether it is the best classification. Firstly, the original data was sorted and ranked, and then, the RSR was calculated:

$${\mathrm{RSR}}_{\mathrm{i}}=\frac{1}{m\times n}\sum_{j=1}^{m}{R}_{ij}$$

Among them, *i* = 1, 2, …, 1542; *j* = 1; *m* represents the number of rows; *n* represents the number of columns; *R*_*ij*_ represents the rank of the element in the *i-th* row and the *j-th* column.

Thirdly, this study calculated the downward cumulative frequency of each group: $\rho =\overline{\mathrm{R}}/\mathrm{n}*100\%$, where $\overline{R}$ is the average rank of RSR, and find its corresponding Probability Unit Probit value according to the “normal allocation law” (The Probability Unit Probit value is used as the independent variable, and the RSR value is used as the dependent variable to calculate the regression equation: $RSR=a+bProbit$. It is used to test whether there is a linear relationship between the dependent variable RSR and the independent variable Probit value).

Fourthly, based on the optimal classifying method [41], this study divided the DOAHPS into five grades: very high, high, general, poor and very poor according to whether* C*_*i*_ < 3; 3 <  = *C*_*i*_ < 4.5; 4.5 <  = *C*_*i*_ < 6; 6 <  = *C*_*i*_ < 7.5; 7.5 <  = *C*_*i*_ and *Probit* < 38; 38 <  = *Probit* < 40; 40 <  = *Probit* < 42; 42 <  = *Probit* < 44; 44 <  = *Probit*.

The T Theil index was used to explain the total difference in within-group and between-group differences, helping to further reflect the causes of unequal allocation of DOAHPS [42]. The specific calculation formula is as follows:

$${\mathrm{T}}_{\mathrm{i}}=\sum_{j}\left(\frac{{r}_{ij}}{{r}_{i}}\right)\times \mathrm{log}\left(\frac{{r}_{ij}/{r}_{i}}{{n}_{ij}/{n}_{i}}\right)$$

$${\mathrm T}_{\mathrm{Between}\;\mathrm{groups}}=\sum_iR_i\times T_i$$

$${\mathrm T}_{\mathrm{Within}\;\mathrm{group}}=\sum_iR_i\times\log\left(\frac{R_i}{N_i}\right)$$

$${\mathrm T}_{\mathrm{Total}}={\mathrm T}_{\mathrm{Between}\;\mathrm{groups}}+{\mathrm T}_{\mathrm{Within}\;\mathrm{group}}$$

Among them,* i* represents the category; *j* represents the number of groups within the category; *T*_*i*_ represents the T Theil index of older adults in each category; *r*_*ij*_ refers to the population in each group (equal to 1 in this study); *r*_*i*_ is the total number of people in category* i*; *n*_*ij*_ is the HPS demands of older adults in each group (in this study = *C*_*i*_); *n*_*i*_ is the sum of the HPS demands assessment scores of older adults in category *i*; *R*_*i*_ is the proportion of older population in each category to the total; *N*_*i*_ is the proportion of DOAHPS’ score in each category to the total score.

### Statistical analysis

This study established an objective evaluation model for DOAHPS using SEM. The Weighted TOPSIS and LR method were used to evaluate the current state and influencing factors for DOAHPS, respectively. The RSR method and the T Theil index were used to analyze the equity level of DOAHPS’ allocation and the main drivers of inequality. Data entry was performed using Epidata 3.1 software. Statistical analysis was performed using Excel 2019, SPSS 20.0 and Amos 20.0 software.

## Results

### Basic information

Among 1,542 study participants, 45.59% were under the age of 70 while 12.97% were equal to or older than 80 years old. Males accounted for 49.55%, married older adults accounted for 74.90%, older adults living in rural areas accounted for 55.97%, and the proportion of older adults who worked in the agricultural sector or others before retirement accounted for nearly 60%. The proportion of older adults with a college degree and above was 7.78%, while more than 60% of older adults obtained primary school education or below. In terms of medical insurance, less than 3% of the population did not purchase basic medical insurance such as employee or resident medical insurance. The analysis found that there were statistical differences in DOAHPS in terms of age, sex, residence, education level, pre-retirement occupation and medical insurance (all *P* < 0.05). See Table 2.

**Table 2:** Characteristics of the study participants (*N* = *1,542*)

Index	Older adults (people (%))	Score^a^$\left(\overline{x}\pm s\right)$	*t/F*	*P*
**Age (years)**				
65 ≤ Age < 70	703 (45.59)	42.43 ± 1.55	2.891	0.021
70 ≤ Age < 75	433 (28.08)	42.67 ± 1.45		
75 ≤ Age < 80	206 (13.36)	42.67 ± 1.45		
80 ≤ Age < 85	147 (9.53)	42.62 ± 1.51		
Age > = 85	53 (3.44)	42.91 ± 1.39		
**Sex**				
Male	764 (49.55)	42.32 ± 1.49	-6.332	< 0.001
Female	778 (50.45)	42.80 ± 1.49		
**Residence**				
Rural	863 (55.97)	41.79 ± 1.27	-27.924	< 0.001
Urban	679 (44.03)	43.55 ± 1.17		
**Education level**				
Illiteracy	360 (23.35)	41.98 ± 1.38	73.678	< 0.001
Primary school	600 (38.91)	42.22 ± 1.42		
Junior high school	305 (19.78)	42.98 ± 1.37		
High school/Technical school	157 (10.18)	43.50 ± 1.44		
College degree and above	120 (7.78)	43.79 ± 1.12		
**Pre-retirement occupation**^b^				
Government staffs	349 (22.63)	43.52 ± 1.30	295.453	< 0.001
Industry and service workers	280 (18.16)	43.52 ± 1.14		
Agricultural sector or others	913 (59.21)	41.91 ± 1.31		
**Marital status**				
Unmarried	25 (1.62)	42.25 ± 1.62	1.563	0.210
Married	1155 (74.90)	42.60 ± 1.50		
Divorced, widowed and others	362 (23.48)	42.47 ± 1.51		
**Medical insurance**^c^				
Basic Medical Insurance for Urban Employees^d^	512 (33.20)	43.57 ± 1.19	223.095	< 0.001
Basic Medical Insurance for Residents^e^	996 (64.59)	42.05 ± 1.39		
Others	34 (2.21)	42.53 ± 1.43		

^a^Score: The score of the demands of older adults for health promotion services (DOAHPS) (0–100); ^b^Pre-retirement occupation: Occupations that older people mainly engaged in before retirement; ^c^Medical insurance: The main type of medical insurance that older adults possess; ^d^Basic Medical Insurance for Urban Employees: In China, it refers to basic medical insurance program mandated by law, in which all urban firms' employees must enroll. The insurance premium shall be borne by both the employer and the employee. ^e^Basic Medical Insurance for Residents: It is a kind of basic medical insurance for residents. Insurance premiums are mainly paid by individual residents (families), supplemented by appropriate government subsidies

### Current state and influencing factors of DOAHPS

#### The current state of DOAHPS

The survey data was substituted into the evaluation model of DOAHPS constructed by SEM in this study, and the Weighted TOPSIS method was used to evaluate the demands. The analysis results showed that the weights of health status, health literacy and behavior, preventive care services utilization were 0.2800, 0.3879 and 0.3321, respectively. Specific to each evaluation dimension, the top three dimensions of the weight coefficient were family doctor, health publicity and health checkup, which were 0.1245, 0.1171, and 0.0905, respectively. The three dimensions with the highest scores included daily activities, behavioral capacity and communication, with scores of 53.79 ± 15.40, 53.04 ± 16.93 and 48.42 ± 14.36, respectively. The analysis also found that the weight rankings and score rankings of the evaluation dimensions were not consistent (Table 3).

**Table 3:** The demands of older adults for health promotion services evaluation results

Indicators	Weight coefficient	Sequence	Score^a^$\left(\overline{x}\pm s\right)$	Sequence
**Health status**	**0.2800**	-		
Behavioral capacity^b^	0.0558	11	53.04 ± 16.93	2
Self-care	0.0541	12	48.23 ± 14.53	4
Daily activities	0.0563	10	53.79 ± 15.40	1
Physical pain	0.0604	9	43.85 ± 14.61	5
Anxiety or depression	0.0534	13	39.34 ± 12.68	8
**Health literacy and behavior**	**0.3879**	-		
Communication^c^	0.0412	14	48.42 ± 14.36	3
Health information	0.0681	6	37.83 ± 21.17	9
Information utilization^d^	0.0651	8	34.12 ± 17.96	12
Smoking	0.0687	5	41.81 ± 21.11	7
Drinking	0.0674	7	43.42 ± 21.35	6
Physical exercise	0.0774	4	37.70 ± 35.14	10
**Preventive care service utilization**	**0.3321**	-		
Health checkup^e^	0.0905	3	34.56 ± 18.16	11
Family doctor	0.1245	1	20.67 ± 19.86	13
Health publicity^f^	0.1171	2	27.98 ± 23.92	14

^a^Score: The score of the demands of older adults for health promotion services (DOAHPS) (0–100); ^b^Behavioral capacity: Refers to the level of behavioral ability such as autonomous walking and recognizing orientation; ^c^Communication: Ease of communication with providers when accessing health or preventive care services; ^d^Information utilization: The extent to which an individual changes his/her own lifestyle as directed by health or wellness information; ^e^Health checkup: Whether to have participated in regular health examinations (non-entry physical examinations, non-disease-related examinations); ^f^Health publicity: Have you received health publicity/guidance provided by primary medical institutions, et al.? (Excluding disease follow-up and follow-up)

#### The influencing factors of DOAHPS

In this study, the LR method was used to analyze the influencing factors of DOAHPS. According to the SEM and univariate analysis results, referring to existing related studies [26, 28], the results of the evaluation of DOAHPS as the dependent variable, the variables age, sex, residence, education level, pre-retirement occupation and medical insurance were included as independent variables in the LR model. In this study, according to the average level of the evaluation of DOAHPS, the evaluation of DOAHPS less than 43 points was scored as 0, and greater than or equal to 43 points was scored as 1. The assignment of the independent variable is shown in Table 4. The R^2^ of the regression model was found to be greater than 0.4, indicating that the model fit was at an acceptable level.

**Table 4:** Variables assignment

Variables	Assignment
DOAHPS^a^	< 43 points = 0, ≥ 43 points = 1
Age	65 ≤ Age < 70 = 1, 70 ≤ Age < 75 = 2, 75 ≤ Age < 80 = 3, 80 ≤ Age < 85 = 4, Age > = 85 = 5
Sex	Male = 0, Female = 1
Residence	Rural = 0, Urban = 1
Education level	Illiteracy = 1, Primary school = 2, Junior high school = 3, High School/Technical School = 4, College degree and above = 5
Pre-retirement occupation	Government staffs = 1, Industry and service workers = 2, Agricultural sector or others = 3
Medical insurance	Basic Medical Insurance for Urban Employees = 1, Basic Medical Insurance for Residents = 2, Others = 3

^a^*DOAHPS* Demands of older adults for health promotion services

The results of LR analysis showed that sex, residence, education level and pre-retirement occupation were the main influencing factors of DOAHPS (*P* < 0.05). Among them, the HPS demands of older adults with no education, primary school and high school/technical school were 0.302, 0.397 and 0.536 times that of older adults with education level of college degree and above, respectively (*OR* = 0.302, *95%CI* = 0.157 ~ 0.581; *OR* = 0.397, *95%CI* = 0.216 ~ 0.728; *OR* = 0.536, *95%CI* = 0.291 ~ 0.987). The HPS demands of older adults who engaged in government and industry and service work as a pre-retirement occupation were 1.707 and 2.422 times higher than those of older adults who engaged in the agriculture sector or others, respectively (*OR* = 1.707, *95%CI* = 1.050 ~ 2.775; *OR* = 2.422, *95%CI* = 1.550 ~ 3.786). The effect of age and the main medical insurance type on DOAHPS was not statistically significant (*P* > 0.05). See Table 5.

**Table 5:** Logistic Regression (*LR*) results

Indicators	*β*	*SE*^*a*^	*Wals*^*b*^	*P*	*OR (95%CI)*
**Sex**	0.767	0.142	29.122	< 0.001	2.154 (1.630 ~ 2.846)
**Age (Age > = 85)**	-	-	8.529	0.074	-
65 ≤ Age < 70	0.349	0.352	0.984	0.321	1.418 (0.711 ~ 2.826)
70 ≤ Age < 75	0.298	0.357	0.696	0.404	1.347 (0.669 ~ 2.715)
75 ≤ Age < 80	0.085	0.378	0.051	0.822	1.089 (0.519 ~ 2.284)
80 ≤ Age < 85	-0.289	0.385	0.564	0.453	0.749 (0.352 ~ 1.593)
**Residence**	1.862	0.212	77.001	< 0.001	6.437 (4.247 ~ 9.757)
**Education level (College degree and above)**	-	-	15.077	0.005	-
Illiteracy	-1.197	0.334	12.854	< 0.001	0.302 (0.157 ~ 0.581)
Primary school	-0.924	0.310	8.898	0.003	0.397 (0.216 ~ 0.728)
Junior high school	-0.572	0.305	3.517	0.061	0.565 (0.311 ~ 1.026)
High school/Technical school	-0.624	0.311	4.011	0.045	0.536 (0.291 ~ 0.987)
**Pre-retirement occupation (Agricultural sector or others)**	-	-	15.115	0.001	-
Government staffs	0.535	0.248	4.650	0.031	1.707 (1.050 ~ 2.775)
Industry and service workers	0.885	0.228	15.091	< 0.001	2.422 (1.550 ~ 3.786)
**Medical insurance (Others)**	-	-	2.708	0.258	-
Basic Medical Insurance for Urban Employees^c^	0.543	0.445	1.493	0.222	1.722 (0.720 ~ 4.118)
Basic Medical Insurance for Residents^d^	0.262	0.448	0.342	0.559	1.300 (0.540 ~ 3.130)
**Constant**	-4.360	0.706	38.096	< 0.001	0.013

^a^: *SE* refers to standard error; ^b^: Wals is a statistic used to test whether the independent variable has an effect on the dependent variable; ^c^Basic Medical Insurance for Urban Employees: In China, it refers to basic medical insurance program mandated by law, in which all urban firms' employees must enroll; The insurance premium shall be borne by both the employer and the employee; ^d^Basic Medical Insurance for Residents: It is a kind of basic medical insurance for residents. Insurance premiums are mainly paid by individual residents (families), supplemented by appropriate government subsidies

### Equity level of allocation of DOAHPS among older adults with different characteristics and its influencing factors

#### The equity level of allocation of DOAHPS

**Fig. 4:**
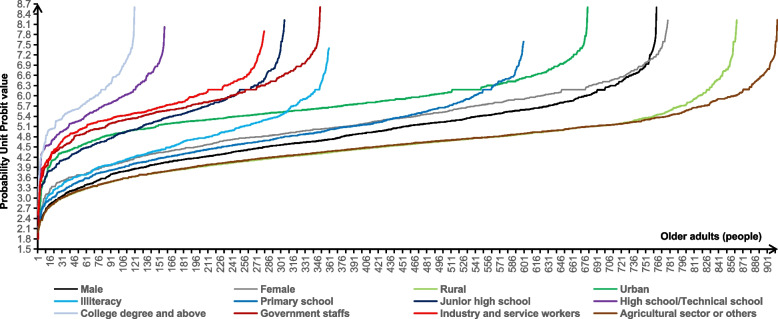
The Probability Unit Probit value of the demands of older adults with different characteristics for health promotion services

In this study, the evaluation results of DOAHPS were ranked from low to high, and were ranked and analyzed using the RSR method. The specific Probability Unit Probit value was shown in Fig. 4, and there is a linear relationship between the RSR value and the Probit value. Based on the evaluation results and Probit value of DOAHPS, the DOAHPS were divided into 5 grades using the classification criteria in the "Methods" section (Table 6). The results showed that the number of older adults with very poor, poor, general, high and very high-level HPS demands accounted for 2.27%, 28.60%, 53.05%, 15.43% and 0.65%, respectively. At the same time, the allocation of HPS demands grades of older adults with different sex, residence, education level and pre-retirement occupation was significantly different (*P* < 0.05). In addition, the level of HPS demands of older adults in different residences and pre-retirement occupations was relatively different.

**Table 6:** Equity analysis of allocation of DOAHPS^a^

Indicators	Grade (People(%))					^χ2^	*P*
	Very poor	Poor	General	High	Very high		
**Sex**							
Male	26 (3.40)	255 (33.38)	393 (51.44)	87 (11.39)	3 (0.39)	38.991	< 0.001
Female	9 (1.16)	186 (23.91)	425 (54.63)	151 (19.41)	7 (0.90)		
**Residence**							
Rural	31 (3.59)	395 (45.77)	389 (45.08)	43 (4.98)	5 (0.58)	379.501	< 0.001
Urban	4 (0.59)	46 (6.77)	429 (63.18)	195 (28.72)	5 (0.74)		
**Education level**							
Illiteracy	12 (3.33)	153 (42.50)	171 (47.50)	24 (6.67)	0 (0.00)	323.431^c^	< 0.001
Primary school	18 (3.30)	211 (35.17)	309 (51.50)	61 (10.17)	1 (0.17)		
Junior high school	2 (0.66)	63 (20.66)	177 (58.03)	58 (19.02)	5 (1.64)		
High school/Technical school	2 (1.27)	7 (4.46)	98 (62.42)	49 (31.21)	1 (0.64)		
College degree and above	1 (0.83)	7 (5.83)	63 (52.50)	46 (38.33)	3 (2.50)		
**Pre-retirement occupation**^b^							
Government staffs	2 (0.57)	29 (8.31)	207 (59.31)	107 (30.66)	4 (1.15)	244.906	< 0.001
Industry and service workers	2 (0.71)	23 (8.21)	171 (61.07)	81 (28.93)	3 (1.07)		
Agricultural sector or others	31 (3.40)	389 (42.61)	440 (48.19)	50 (5.48)	3 (0.33)		
**Total**	35 (2.27)	441 (28.60)	818 (53.05)	238 (15.43)	10 (0.65)	-	-

^a^*DOAHPS* Demands of older adults for health promotion services; ^b^Pre-retirement occupation: Occupations that older people mainly engaged in before retirement; ^c^: Because the number of people choosing "very high" for a single category was < 5, the analysis results shown were the result of combining items

#### Influencing factors of DOAHPS’ equity level

The results of the analysis showed that the total T Theil index of DOAHPS was 2.7433*10^–4^. The contribution rate of intra-group differences was greater than that of inter-group differences, exceeding 72%, indicating that the differences in DOAHPS were mainly caused by inter-group differences. The analysis results by sex and residence showed that the total T Theil index of each group was 1.6462*10^–4^-2.6842*10^–4^, and the total T Theil index of females was higher than that of males, and the total T Theil index of older adults in rural areas was higher than that in urban areas. In rural and urban areas, the contribution rate of the intra-group T Theil index to the grading differences in DOAHPS exceeded 97%. In terms of pre-retirement occupation, the T Theil index within the group contributed less than 70% to the total T Theil index. See Table 7.

**Table 7:** T Theil Index and contribution rate (%) of DOAHPS^a^

Indicators	T Theil index (contribution rate)	Total (10^–4^)	Sex (10^–4^)		Residence (10^–4^)	
			Male	Female	Rural	Urban
Education level	T Total Theil Index	2.7433	2.6842	2.6647	1.9806	1.6462
	T Theil index within group (%)	2.3096 (84.19)	2.0909 (77.90)	2.2029 (82.67)	1.9229 (97.08)	1.6255 (98.74)
	T Theil index between groups (%)	0.4336 (15.81)	0.5933 (22.10)	0.4618 (17.33)	0.0577 (2.92)	0.0207 (1.26)
Pre-retirement occupation^b^	T Total Theil Index	2.7433	2.6842	2.6647	1.9806	1.6462
	T Theil index within group (%)	1.9930 (72.66)	1.8327 (68.28)	1.9967 (74.93)	1.9304 (97.46)	1.6255 (98.74)
	T Theil index between groups (%)	0.7499 (27.34)	0.8514 (31.72)	0.6680 (25.07)	0.0502 (2.54)	0.0207 (1.26)

^a^*DOAHPS* Demands of older adults for health promotion services; ^b^Pre-retirement occupation: Occupations that older people mainly engaged in before retirement

## Discussion

The purpose of this study was to construct a model and objectively evaluate the current state and equity level of allocation among the population of DOAHPS by analyzing data from the survey on the DOAHPS in eastern, central and western China, as well as to identify the most influential factors that affect the current state and equity level of DOAHPS. The findings of the analysis showed that health status and health literacy and behavior were positively correlated to DOAHPS (*r* = 0.40, 0.38). The main factors influencing DOAHPS were sex, residence, education level and pre-retirement occupation. Results of RSR analysis showed that the proportion of older adults with low-level HPS demands was greater than the proportion of older adults with high-level demands, and the allocation inequity was mainly affected by the factors within the group (the contribution rate of T Theil index within group > 72%).

Specifically, compared with complete demands, the total evaluation score of DOAHPS was 42.57 ± 1.51, and health status, literacy and behavior were positively associated with DOAHPS. SME analysis indicated that health status was considered the factor that is mostly associated with DOAHPS. The relatively low correlation between future health promotion demand and current utilization of preventive care services was consistent with other studies [43]. On the other hand, both health status and preventive care services utilization had a direct impact on DOAHPS. While health literacy and behavior have a direct impact on DOAHPS, this study found that it also has an indirect effect on DOAHPS with health status and preventive care services utilization. The negative relationship between health literacy and behavior and preventive care services utilization may be related to the fact that good health literacy and behavior leads to a positive self-health evaluation, which reduces the motivation for preventive health services utilization among older adults, and that the clear perception of self-health status resulting from preventive health services utilization also tends to reduce the motivation for preventive health services utilization. At the same time, consistent with the SEM analysis, the results based on the Weighted TOPSIS method showed that older adults had relatively the best evaluation of their health status [10]. However, the weights of each evaluation factor show a basically opposite trend to evaluation results. Older adults are very concerned about their immediate health status, and the evaluation is relatively good and consistent, but the utilization of preventive care services by older adults is low and the difference is significant, and hence the amount and equity of utilization need to be further improved [13].

The results also showed that the total HPS demands of female participants were higher than that of males. The HPS requirements of urban participants were more than those of rural ones, and the disparity between urban and rural locales was bigger than that between gender. A study by Astrid E et al. showed similar findings [44]. On the one hand, the higher education level of older adults favors DOAHPS, and this effect increases as the education level reaches high school/technical school. With the continuous reduction of education level, the DOAHPS remained basically stable [28]. This may be related to the fact that Chinese students study the basic knowledge of life and society below high school/technical school, while university education focuses on the cultivation of professional knowledge and self-learning ability [45]. The current study also found that HPS demands were relatively lowest among older adults who were engaged in agricultural activities before retirement. One possible explanation is that, on average, farmers in developing countries have lower economic and political status.

In addition, this study found that the allocation of DOAHPS was generally equitable among the population, but the beneficiaries were more likely to be older urban females. And in terms of education and pre-retirement occupations, this trend is mainly due to differences within groups of different education levels and pre-retirement occupation categories. This finding is similar to the study by Gallo HB et al., which established a positive relationship between the social hierarchy of the population and the ability to prevent HPS demands [46]. One probable explanation is that older persons in the top societal class tend to have a higher level of education or a better economic standing, greater understanding and purchasing power of HPS, and a higher HPS utilization rate. However, older adults with low social stratification lack the time and energy to actively carry out HPS, and the adoption rate of HPS behaviors is also low [27]. Given that sex is an unmodified variable, policymakers and organizers should prioritize the HPS demands of older rural men who face intellectual, psychological, or economic constraints.

Finally, the unequal allocation of DOAHPS among different groups is mainly attributable to within-group differences, which is a reminder that DOAHPS is simultaneously influenced by multiple confounding factors, and no single characteristic alone determines an individual's total demand [31, 47, 48]. More attention should be paid to the inequality of demands within older adults with the same personal characteristics, such as sex, pre-retirement occupation, education level and others [32]. Therefore, the authors advise that when the government formulates policies such as the provision and evaluation of relevant HPS, it could pay greater consideration to the individual capacity disparities of older persons as opposed to developing classification standards purely based on residence and income.

This study had several strengths: Firstly, the authors focused their study on an objective evaluation of DOAHPS. Secondly, this study used the RSR method to analyze the equity of the allocation of DOAHPS among the older adult population. Thirdly, the analysis results based on the T-Theil index can provide an evidence-based reference for understanding and strengthening the equity of the allocation of DOAHPS. A significant limitation of this study, however, is that the health promotion-based design did not include medical services and did not consider possible immunization demands in older adults. Furthermore, methods such as the RSR employed in this study have strict requirements for the quantity and quality of data, limiting their applicability in other similar surveys. Thirdly, given the cross-sectional nature of the current study, the authors believe that there may be other important long-term changes, such as socioeconomic development and changes in health concepts, which deserve further exploration and analysis.

## Conclusions

This study objectively evaluated the current state and equity level of allocation among older adults of DOAHPS in China, and explored the main factors affecting DOAHPS’ current state and equity level. The results showed that, compared with the maximum level, the total DOAHPS level was found to be moderate, but the demands of educated urban older adults may be relatively greater. The observed inequities in the allocation of DOAHPS were primarily related to differences in education level and pre-retirement occupation within groups. To better address HPS for older adults, policymakers could target older males with low levels of education who reside in rural regions, as well as those who reside in urban areas without a steady employment history.

## Data Availability

Availability of data supporting the findings of this study is limited and therefore not publicly available. Data are however available from the corresponding author upon reasonable request.

## Abbreviations

DOAHPS: Demands of older adults for health promotion services
HPS: Health promotion services
SEM: Structural Equation Modeling
LR: Logistic regression
RSR: Rank Sum Ratio
TOPSIS: Technique for Order Preference by Similarity to Ideal Solution
